# 

**Published:** 1848-01

**Authors:** 


*Bartlett on Fevers.*—We have received the new edition of this work, and hope soon to be able to give it that careful examination which it merits.

